# Identifying and Managing Malnutrition, Frailty and Sarcopenia in the Community: A Narrative Review

**DOI:** 10.3390/nu13072316

**Published:** 2021-07-05

**Authors:** Shelley Roberts, Peter Collins, Megan Rattray

**Affiliations:** 1 School of Health Sciences and Social Work, Griffith University, Gold Coast 4222, Australia; m.rattray@griffith.edu.au; 2 Menzies Health Institute Queensland, Griffith University, Gold Coast 4222, Australia; 3 Allied Health Research, Gold Coast Hospital and Health Service, Gold Coast 4219, Australia; 4 Dietetics and Food Services, Mater Health, Brisbane 4101, Australia; peter.collins@mater.org.au; 5 Mater Research Institute, University of Queensland, Brisbane 4101, Australia

**Keywords:** malnutrition, frailty, sarcopenia, community, older adults, nutrition care

## Abstract

Malnutrition, frailty and sarcopenia are becoming increasingly prevalent among community-dwelling older adults; yet are often unidentified and untreated in community settings. There is an urgent need for community-based healthcare professionals (HCPs) from all disciplines, including medicine, nursing and allied health, to be aware of, and to be able to recognise and appropriately manage these conditions. This paper provides a comprehensive overview of malnutrition, frailty and sarcopenia in the community, including their definitions, prevalence, impacts and causes/risk factors; and guidance on how these conditions may be identified and managed by HCPs in the community. A detailed description of the care process, including screening and referral, assessment and diagnosis, intervention, and monitoring and evaluation, relevant to the community context, is also provided. Further research exploring the barriers/enablers to delivering high-quality nutrition care to older community-dwelling adults who are malnourished, frail or sarcopenic is recommended, to inform the development of specific guidance for HCPs in identifying and managing these conditions in the community.

## 1. Introduction

Australia and New Zealand (ANZ) have ageing populations, with the number of people aged ≥ 80 years expected to increase by >200% by 2050, totalling > 510,000 New Zealanders and 2.8 million Australians in this age group within the next 30 years [1,2]. This is likely to result in an increased prevalence of age-associated conditions such as protein-energy malnutrition, frailty and sarcopenia; common and overlapping problems impacting the functional and health outcomes of older adults that are often left unidentified and untreated in community settings [3]. Consequently, these conditions lead to increased healthcare costs, with malnourished, frail and/or sarcopenic individuals requiring more health care professional (HCP) consultations, hospitalization, health care monitoring and treatments [4,5]. While these conditions are traditionally recognised and treated in hospital, not all community-dwelling adults are hospitalised; and being a short period in a person’s life, hospitalisation is usually insufficient to correct malnutrition or associated conditions. As patients are acutely unwell while hospitalised, it may also be difficult for them to engage in care discussions or implement nutrition advice. Therefore, there is an urgent need for HCPs from all disciplines, including medicine, nursing and allied health, to understand, recognise and act on suspected malnutrition, frailty and sarcopenia among community-dwelling older adults. This review will focus on malnutrition, frailty and sarcopenia in the ANZ community, covering their prevalence, impacts and risk factors; as well as the steps for identifying and managing these conditions in this setting.

## 2. Methods

We conducted a narrative review to synthesize and integrate research published to date on the topics of malnutrition, frailty and sarcopenia, in order to describe the problem and identify possible management strategies [6]. Several databases were searched to identify relevant studies: Google Scholar, Medline, PubMed, and Scopus. Search terms such as “malnutrition”, “frailty”, “sarcopenia”, “community”, “older adults”, “nutrition care”, “Australia”, “New Zealand” and “review” were used in various combinations to capture relevant studies. Forward and backward citation tracking was also performed on relevant literature to maximize retrieval of eligible evidence. Our focus was on summarizing original evidence reported by peer-reviewed Australian and New Zealand studies considering the contextual nature of disease and treatment. However, relevant published reviews synthesizing international literature in this area were also captured to improve the robustness and generalizability of our conclusions. A date restriction (published ≥ 2010) was in place to capture and summarize the evidence for prevalence literature only. Restrictions were not imposed on study design or study quality. The six items of the scale for quality assessment of narrative review articles (SANRA) were used as a guide to inform our methodological approach and to report the findings [7].

## 3. The Concepts of Malnutrition, Frailty and Sarcopenia

### 3.1. Protein-Energy Malnutrition

Protein-energy malnutrition (PEM) is common among elderly adults and people with chronic illness. It is a result of insufficient energy and protein intake to meet the body’s needs, causing weight loss and wasting of muscle and fat stores [8]. Historically, there is a lack of consensus on the definition of malnutrition and which clinical indictors are important for its identification. Consequently, international nutrition societies such as the European Society for Parenteral and Enteral Nutrition (ESPEN) [9] and The American Society for Parenteral and Enteral Nutrition (ASPEN) [10] have developed their own sets of criteria for diagnosing malnutrition. While both bear similarities, the clinical indictors used to operationalise PEM differ. Recently, the Global Leadership Initiative on Malnutrition (GLIM) criteria were developed in an attempt to standardise the diagnosis of malnutrition so that its prevalence, interventions and outcomes can be compared internationally [11]. Despite their differences, all three methods recognise PEM as a syndrome resulting from a lack of uptake and/or intake of nutrition, leading to altered body composition, attenuated physical and mental function, and poorer clinical outcomes [12].

### 3.2. Frailty 

Frailty is a state of diminished physiological reserve, where the body is unable to resist minor stressors, leading to adverse health outcomes [13]. Pre-frailty, which represents an intermediate level of elevated vulnerability, refers to the transitional state between robustness and frailty [14]. Not everyone will develop frailty, and an individual’s severity or risk status can change [15]. The Fried Frailty Phenotype (FFP) and the cumulative deficits approach are two common approaches used to define frailty [16]. The former is characterized by the presence of three or more physical characteristics (weakness, decreased endurance, slow performance, exhaustion, weight loss) that are often (but not completely) unique and separate from comorbidities and disability alone [13], while the latter approach views frailty as related to the accumulation of various deficits (e.g., mental, social, and physical factors), rather than a one-dimensional set of criteria [17]. The predictive validity for adverse health outcomes has been shown for both approaches [18].

### 3.3. Sarcopenia 

Sarcopenia is an age-related skeletal muscle disorder characterized by loss of lean mass and function [19,20]. Chronic inflammation, loss of α-motor neurons, redox imbalance, altered hormonal status and myocyte autophagy, muscular mitochondrial dysfunction, accelerated apoptosis of myonuclei and impaired satellite cell function are considered the major factors contributing to age-related muscle wasting. Given many of these factors are associated with ageing, sarcopenia is often framed as a geroscience condition [21]. There are several widely employed definitions for sarcopenia including those from European Working Group on Sarcopenia in Older People (EWGSOP1, released in 2010 [22]; and EWGSOP2 released in 2019 [23]) and the US Foundation for the National Institutes of Health (FNIH) [20]. The EWGSOP1 definition uses an algorithm comprising of gait speed, muscle mass and handgrip strength, with cut-off points dependent upon individuals’ demographics to determine sarcopenia severity, with an emphasis placed on muscle mass [22]. This definition was recently updated (EWGSOP2) to replace muscle mass with muscle strength as the primary indicator for probable sarcopenia [23]. Alternatively, the FNIH definition uses a data-driven process, derived from several cohorts of community-dwelling older persons, to identify criteria for clinically relevant weakness (i.e., low grip strength), slowness (i.e., low gait speed) and low appendicular lean mass [20]. Sarcopenia can be identified as low muscle mass with low grip strength using the FNIH definition [20], or slowness with weakness and low lean mass (for severe sarcopenia) as per EWGSOP definitions. Although these three definitions use different strategies and cut-off points, they all recognise that the measurement of muscle mass and quality is the confirmatory step to indicate the presence of sarcopenia.

### 3.4. Overlap between Malnutrition, Frailty and Sarcopenia

Although PEM, frailty and sarcopenia are distinct from each other, there is overlap and synergy between the conditions [24]. For example, frailty and sarcopenia are related syndromes, sharing features in common such as lower lean mass and reduced physical function [22]. Malnutrition plays a key role in the pathogenesis of both these conditions and vice versa [25], demonstrating the complex and synergistic relationship by which each condition is accelerated by the next. 

## 4. Prevalence of Malnutrition, Frailty and Sarcopenia

The prevalence of malnutrition among ANZ community-dwelling older adults ranges between 1% and 17%; and between 4% and 63% are at risk for developing malnutrition [26,27,28,29,30,31,32,33,34,35,36,37] (refer to Table S1 in Supplementary Files). Several tools were used to assess malnutrition in studies, with the modified Seniors in the Community: Risk Evaluation for Eating and Nutrition questionnaire (SCREEN© II) among the most common. The use of different tools, coupled with the population studied, may explain the differences in malnutrition prevalence reported across studies. Indeed, a recent study that used secondary data analyses from 11 European and one New Zealand study encompassing over 5000 older adults demonstrated higher malnutrition rates in adults > 80 years, in women, and in those with one or multiple morbidities; and prevalence differed by geographic location and tool employed [38]. The criteria used to identify malnutrition appear to strongly affect prevalence, highlighting the importance of considering each criterion separately, as each may indicate a nutritional problem. 

Prevalence of frailty and pre-frailty among ANZ community-dwelling older adults is estimated to be between 2–29% and 41–54%, respectively [16,39,40,41,42,43,44] (Table S1). Frailty rates appear higher among Australian women (18–29%) than men (6–21%). While the Modified Fried Frailty Phenotype (FFP) appears to be the most common approach, a number of tools were used to assess frailty. This, in conjunction with the population screened (e.g., men to women ratio), may help explain differences in findings between studies. In fact, one study reported minimal agreement between four different frailty measures; 35% of participants were identified as frail by only one measure, 9% by two measures, 3% by three measures, and <1% by all four measures [43], illustrating how the criteria or tools used to identify frailty can greatly impact its diagnosis. 

The prevalence of sarcopenia is estimated between 1% and 24% among community-dwelling older Australian adults and appears to vary depending on the diagnostic tool used [44,45,46,47,48,49] (Table S1). Two studies explored the level of agreement between various tools within their participant sample; Sim et al. (2019) found four sarcopenia definitions differed substantially: FNIH (9%), FNIH2 (12%), EWGSOP1 (24%) and EWGSOP2 (11%) [46]. Meanwhile, Sui et al. (2021) found moderate agreement between EWGSOP1 and EWGSOP2, and poor agreement between FNIH and EWGSOP1/EWGSOP2 [47,48]. Poor agreement between EWGSOP1 and FNIH is well documented [50,51]. The prevalence of frailty and sarcopenia in combination was explored in only one study, which demonstrated that people with frailty are likely to have sarcopenia, but not all people with sarcopenia are frail [44]. International studies have reported similar findings [52,53].

## 5. Impact and Effects of Malnutrition, Frailty and Sarcopenia

Malnutrition, frailty and sarcopenia have serious consequences at an individual and societal level. Malnutrition adversely impacts the body’s healing process and increases a person’s risk of functional decline [54], infections [55], falls [56,57], pressure injuries [58], hospitalisation [35,54], institutionalization [54] and mortality [35]. In turn, these adverse events negatively influence an individual’s quality of life, especially in elderly persons, and that of their families, and substantially contribute to acute and long-term healthcare costs [59]. Similarly, the consequences of frailty and sarcopenia include increased risk of falls and fractures [60], disability [45,60,61], poor quality of life [62,63], and mortality [44,45,64]. Importantly, a recent study reported that frailty and sarcopenia in combination are over three times more predictive of mortality than either condition alone in older community-dwelling adults [44]. At a societal level, frailty and sarcopenia lead to higher costs [4,5] and increased use of health care resources, such as HCP consultation/monitoring [5], emergency department visits hospitalization [65], and institutionalisation [45]. Although cost estimates for the Australian population are not available, increasing life expectancy indicates that frailty, sarcopenia and malnutrition are a growing economic burden. 

## 6. Causes/Risk Factors of Malnutrition, Frailty and Sarcopenia

Causes of PEM, frailty and sarcopenia in older community-dwelling adults are multifactorial and interrelated (Table 1). While many factors have been correlated with PEM in community-dwelling older adults, and are thus suspected to be determinants of malnutrition, much of this evidence has been generated from studies with limited ability to make causal inferences. As a result, contradictory findings have been reported. For example, a recent review of 35 studies reported conflicting evidence for dental status, swallowing, cognitive function, depression, polypharmacy, constipation and periodontal disease being determinants of PEM in older adults [12]. Despite this, moderate to strong evidence for an association with PEM have consistently been reported for several modifiable and non-modifiable determinates, including poor appetite [12,66,67], hospitalization [12,31,67], poor self-reported health [12,66,67] and increasing age [31,36,66]. Further, moderate quality evidence suggests chewing difficulties, mouth pain, gum issues, visual and hearing impairments, smoking status, alcohol consumption and physical activity levels, complaints about taste of food and specific nutrient intake are not determinants of malnutrition [12].

Similarly, several modifiable and non-modifiable determinates are consistently associated with the development of frailty and sarcopenia. Nutrition is acknowledged as a major modifiable risk factor in the context of frailty and sarcopenia. Five previous systematic reviews have been conducted on the association between nutrition and frailty [68,69,70,71] or sarcopenia [73]. Further, international studies have reported that malnutrition is associated with a fourfold and fivefold higher risk of developing sarcopenia [78] and frailty [79], respectively. Several factors might be responsible for this close relationship, including oral health [71], nutritional status [68], diet quality [68,73], the antioxidant capacity of the diet [68,70] and protein intake [68,69]. Of interest, the psychological and social factors impacting frailty and sarcopenia have been studied to a limited extent. Lastly, non-modifiable determinants such as increasing age is associated with the development all these conditions [15,16,31,36,44,47,66,80], while evidence for the role of gender remains contradictory. 

## 7. Addressing Malnutrition, Frailty and Sarcopenia Using the Nutrition Care Process

The Nutrition Care Process (NCP) is a standardised model used to guide HCPs in providing consistent and high-quality nutrition care [81]. It involves four main steps: (1) nutrition assessment and re-assessment, (2) nutrition diagnosis, (3) nutrition intervention, (4) nutrition monitoring and evaluation, as well as (a) precursor (screening and referral) and (b) follow-up (outcomes management) steps [81]. While this process is specific to nutrition care, frailty and sarcopenia are closely related to and intertwined with malnutrition; hence, we propose that the NCP steps can be applied to all three conditions, using tools and approaches appropriate to each. This section will outline the process for identifying and managing malnutrition, frailty and sarcopenia in the community.

### 7.1. Screening and Referral 

The importance of identifying malnutrition, frailty and sarcopenia in the community is clear when considering their impacts on patients and health services. Screening is a preceding step to the NCP that allows HCPs to recognise and prioritise at-risk patients so they can be formally assessed and managed or referred to other HCPs. It involves using a validated screening tool to determine a patient’s risk of having one or more of these conditions. There are several valid and reliable tools available for use in clinical and community settings (see Table 2). Screening tools can be used by any HCP and should be selected based on their practicability and reliability in the intended setting [3]. Screening tools are readily available online; however, if a practice setting does not use formal screening tools routinely, HCPs can use informal methods (such as recognising risk factors; Table 1) to identify and prioritise at-risk patients for referral to appropriate HCPs.

While risk screening is typically conducted in clinical settings (for example, most Australian and New Zealand hospitals mandate nutrition risk screening using a validated tool), screening in the community is equally important, as not all people will be admitted to hospital. General practitioners (GPs), practice nurses and community-based allied health clinicians are all well-placed to screen for malnutrition, frailty and sarcopenia. Screening should be prioritised for those at increased risk, such as patients with acute or chronic disease, reduced BMI or recent unintentional weight loss, older age or other risk factors (per Table 1). Patients identified as at risk should be referred to accredited HCP/s for formal assessment (i.e., dietitian for malnutrition risk; physiotherapist/exercise physiologist for frailty/sarcopenia risk).

Patients themselves can and should be involved in the screening process. Literature suggests that patients desire to be involved in their nutrition care [118,119], and several screening tools have been validated for use by patients and/or family members. A Dutch study found that online self-screening for malnutrition may be an easy, useful, accessible and contemporary way to identify older adults at nutritional risk in the community [120]. Consistent with a patient-centred care approach, patients should be adequately informed about the purpose and outcome of risk screening. A 2020 systematic review found that while most patients were accepting of nutrition screening, many did not understand its purpose or its results, which caused some to disbelieve or disregard their risk and was a barrier to patients enacting dietary advice [121]. This highlights the importance of engaging patients in the care process and keeping them informed to make health care decisions.

Community-based HCPs can provide better quality and more holistic care by using validated screening tools or identifying risk factors among community-dwelling adults who may have or be at risk of malnutrition, frailty and sarcopenia; so appropriate interventions or referral to other HCPs (such as dietitians) can be enacted [3]. Table 2 provides an overview of screening tools used in the community setting to identify patients at risk of malnutrition, frailty and sarcopenia.

### 7.2. Assessment and Diagnosis

Nutrition assessment involves systematically collecting and evaluating relevant information to diagnose nutrition-related problems and understand their causes [81]. This is typically done by a dietitian and involves evaluating a patient’s nutrition intake, anthropometry, biochemistry, clinical signs/symptoms, and nutrition-focused physical findings (Table 3). Dietitians use this information, along with formal nutrition assessment tools, to make a nutrition diagnosis and to inform nutrition interventions. Several nutrition assessment tools exist and are commonly used in clinical and community settings. For example, the Subjective Global Assessment (SGA) can reliably identify malnutrition-related muscle dysfunction and impaired functional status among hospital patients [122]. The Mini Nutritional Assessment (MNA) is designed for and validated in elderly patients (including in community) [123,124,125] and assesses food intake, weight loss, mobility, disease/stress, BMI and neuropsychological problems. It has also been proposed as a useful tool to identify frail patients [93]. While such tools are commonly used in community, there is limited evidence for their diagnostic accuracy in this setting. In fact, one review found that no nutrition assessment tool had undergone sufficient validity testing in community-living adults, and authors were unable to recommend any one tool for diagnosing malnutrition in this group [125]. The best evidence shows moderate confidence in the ability of the MNA to predict death and limited confidence in its ability to predict physical dysfunction [125]. Despite this, it is suggested that nutrition assessment should be undertaken to ensure malnourished patients are adequately managed; and that more diagnostic accuracy studies are needed for all nutrition assessment tools in community settings [125]. Given this, community-based HCPs should refer patients at-risk of, or with suspected malnutrition, to a dietitian for formal assessment (where resources allow); or alternatively, use the domains in Table 3 to make an informal assessment to guide care.

Frailty assessment is still evolving, likely due to the lack of consensus on a universal definition of frailty and how to identify it. There are two models by which frailty is recognised; the Fried Frailty Phenotype defines it as a set of physical characteristics (weakness, exhaustion, weight loss slowness, poor endurance) that are independent of disability and comorbidities [13], while the accumulation of deficits model considers summative physical, mental and social deficits, rather than a specific set of criteria [17]. Further, there seems to be poor distinction between frailty screening and assessment tools in the literature, with tools often used interchangeably. There are also fewer validity, consensus and systematic review papers published on frailty tools (compared to malnutrition tools), making it difficult to compare different tools’ performance in various settings [3]. Systematic reviews have been unable to recommend specific tools for frailty assessment in community-dwelling adults, stating more research is needed [3,126,127]. One author suggests a two-step approach to frailty assessment; (1) a simple self-report screening questionnaire to identify those who would benefit from (2) a further complex assessment [127]. This may help to prioritise patients who are most at-risk and inform care planning. Frailty measures can include HCP judgment-based assessments, physical performance tests (e.g., gait speed, grip strength), physical frailty measures (e.g., frailty phenotype), multi-dimensional instruments (that include other dimensions such as cognition), and frailty indices [128]. Frailty tools should cover one or more of the assessment domains listed in Table 3.

Similar to frailty, the assessment and diagnosis of sarcopenia is an evolving area. However, multiple criteria exist for diagnosing sarcopenia, including the European Working Group on Sarcopenia in Older People (EWGSOP) [22], the International Working Group on Sarcopenia (IWGS) [115], the Asian Working Group for Sarcopenia (AWGS) [116], and the Society for Sarcopenia, Cachexia and Wasting Disorders (SCWD) [117]. The Australia and New Zealand Society for Sarcopenia and Frailty Research encourages the adoption of EWGSOP2 definition of sarcopenia in practice [129].

Assessment should occur upon initial referral of patients suspected to be malnourished, frail or sarcopenic; and should be repeated as appropriate to evaluate the effects of interventions (refer to Monitoring and Evaluation section) [81]. 

### 7.3. Intervention

As nutrition intake is a major modifiable risk factor in the development and progression of malnutrition, frailty and sarcopenia, interventions targeting these conditions frequently use nutrition-based strategies that aim to improve an individual’s nutritional status. Table 4 illustrates the use of such interventions among community-dwelling older adults in Australia and New Zealand. These broadly fall within two categories: strategies to influence patient knowledge/behaviour; and strategies to improve dietary intake. While limited intervention studies using nutrition-based strategies among frail or sarcopenic individuals have been conducted in the ANZ context, international work supports the role of nutrition used alone [130,131] or in combination with exercise to aid in the management of these conditions [130,131,132,133]. Further, the same strategies can be applied to treat frailty and sarcopenia as the principle remains the same - to improve an individual’s nutritional status. Importantly, while a combination of nutrition and exercise strategies are recommended to manage/treat malnutrition, frailty and/or sarcopenia, given the interplay of these three conditions, the focus and type of strategies used may vary depending on the primary condition being treated. For example, if the condition being treated is malnutrition, the HCP’s primary focus may be on improving the individual’s nutrition status through the use of nutrition-based strategies, whereas exercise-based strategies may be the focus of interventions for someone being treated for sarcopenia, considering sarcopenia is thought to occur regardless of energy balance [134]. Prior to delivering any nutrition- or exercise-based intervention(s), appropriately trained HCPs should be consulted first (dietitians for nutrition interventions; physiotherapists for exercise interventions) to ensure patient safety and the delivery of high-quality care.

#### 7.3.1. Strategies to Influence Knowledge and Behaviour 

Delivery of one-on-one counselling [135,137,140] and resource kits [136] are common educational strategies used in interventions to improve an individual’s malnutrition or frailty status (Table 4). These strategies align with Australian and European guideline recommendations suggesting that patients and caregivers should be offered knowledge about their nutritional problems and treatment options to promote appropriate nutritional care [141,142]. A large Australian study (*n* = 1145) reported that 82% of at-risk and malnourished community dwelling older adults who received one-on-one individualized counselling had improved PG-SGA scores after six months of intervention [135]. Similarly, a smaller Australian study (*n* = 68) provided older adults with tailored individual dietary advice two weeks post-hospital discharge, and reported that the proportion of at-risk and malnourished adults reduced from 62% to 24% three months after the intervention [137]. For both interventions, education was tailored to the individual’s needs and delivered frequently, at home, by trained dietitians; factors which have been shown to contribute to high adherence to dietary advice among elderly individuals [143]. Despite these positive findings, 55% of at-risk or malnourished patients who were offered free dietetic counselling in the aforementioned study declined to partake [135]. 

This emphasises the difficulty in engaging this high-risk group and suggests the need for nutrition education to be given at opportune times (e.g., by nurses or GPs when elderly adults visit healthcare services). Indeed, there is evidence that nutrition advice delivered by nurses can positively influence the functional outcomes and diets of older people living at home [144]. Further, there is moderate evidence to support the role of domiciliary carers in implementing malnutrition risk screening, education and referral pathways, which may be effective and cost-efficient strategies to manage malnutrition in community-dwelling older adults [145,146]. This suggests strategies to improve nutrition knowledge should not only be limited to dietitians, but also include carers, nurses and GPs in the community. Further, geographically relevant resource kits (which include educational material) have been shown to be effective at improving nutritional status among community-dwelling ANZ adults [136] and should therefore be considered when individual dietary counselling from trained HCPs is not immediately available. 

#### 7.3.2. Strategies to Influence Intake 

The use of ONS is a common strategy used to improve nutrition intake among adults [137,139,140]. These are nutritionally complete drinks that contain a mix of macro- and micro-nutrients. Randomised Control Trials (RCTs) have shown a positive effect of daily ONS consumption on energy/ protein intakes and nutritional status among malnourished older patients after discharge from hospital [147,148]. Yet, the success of such interventions relies on patient adherence. One systematic review reported high compliance to ONS among community-living adults [149]; however others have reported lower [150] or variable adherence [151]. Other influencing factors commonly cited include duration of usage [152], if the patient is aware of reason for ingestion [153], variety of supplements prescribed [149] and how the supplement is taken [149]. As such, education should be provided on the potential benefits of ONS ingestion considering dietary counselling combined with ONS is the most effective intervention to improve energy intake and body weight among older adults [154]. However, other strategies to influence intake should be prioritised where barriers to using ONS have been identified.

Food-based fortification (also called dietary enrichment) is an alternative strategy to improve energy and protein intake [136,138]. This involves increasing the energy and protein of a meal without increasing volume, by adding extra-high-energy/-protein ingredients such as oils, butter, cream, or powdered milk; and/or powdered modules such as casein, whey protein, or maltodextrin. Indeed, a recent systematic review demonstrated that energy- and protein-based fortification can be employed as an effective and well-tolerated intervention to improve dietary intake amongst older adults [155]. This strategy may be particularly useful for patients who do not tolerate or cannot afford ONS and has the advantage of better catering to individual dietary habits and preferences. Low dairy intake has also been identified as a frequently occurring risk factor among at-risk and malnourished community-living older adults [32,137], highlighting another avenue for low-cost dietary intervention. 

Home delivery meal services, such as Meals on Wheels (MOW), which deliver healthy meals to clients’ homes, may also be used as a convenient strategy to improve intakes among individuals who are unable to prepare meals or shop for themselves [137,138]. A recent systematic review reported a beneficial effect of home-delivered meals on intakes of energy, protein and/or micronutrients (e.g., calcium, vitamin A, B complex vitamins, vitamin D, zinc, magnesium and others) in older community-dwelling adults [156]. While evidence shows that meals delivered by this service provide an important contribution to an individual’s overall intake [157], the inclusion of other foods (e.g., snacks) is still necessary for older individuals to meet their nutrition needs; something that is not always acknowledged among these clients [138]. Strategies therefore still need to be tailored amongst MOW clients, given their potentially limited ability to access, cook and/or prepare food. 

### 7.4. Monitoring and Evaluation (Including Documentation)

Thorough monitoring and evaluation are helpful to track patients’ progress and improve their outcomes by identifying any personal and/or environmental factors that may hinder compliance to nutrition intervention [158]. Evidence-based practice and patient preferences should be used to guide the selection of appropriate outcome measures/indicators to assess nutrition care [158]. Unfortunately, an internationally standardized set of outcomes for nutrition care is unavailable [158]; but outcomes to monitor/evaluate nutrition interventions can be classified into the four categories outlined in Table 5 [159,160]. Recent studies on the outcomes used in usual dietetic practice in Australia are lacking, however a 2008 Australian study found dietitians used a range of outcome measures in practice and these aligned with three of the four categories described in Table 5 (healthcare utilisation/cost savings were not routinely assessed, consistent with other studies) [159].

Patients should be involved in selecting the outcomes most important to them. An Australian study identified 11 quality indicators of dietetic services from the perspectives of malnourished older patients, spanning three domains: structure (healthcare systems/environments), processes (dietitian-patient interaction) and outcomes (desired measurable outcomes of nutrition care) [163]. Outcome measures most important to patients were: (1) improvement in health status; (2) improvement or maintenance of independence; and (3) weight gain. Further, a qualitative study examining patients’ experiences with dietetic consultations in the United Kingdom found that patients preferred dietitians who adopted a patient-centred approach and considered what patients wanted from the consultation [162]. As such, HCPs should monitor/evaluate nutrition interventions using a combination of indicators from the four categories outlined in Table 5, factoring in indicators most important to patients, to provide evidence-based, patient-centred care. This requires high level critical reasoning and should be planned thoroughly, also taking into account available data collection tools/methods and feedback strategies to other HCPs.

The frequency of measurement and strategy for data collection/reporting are important aspects of monitoring and evaluation. Data should be collected during the first consultation, halfway through the intervention or when significant changes occur (in adherence, clients’ status or situation) and at the end of the process. However, it is often up to the judgement of the HCP, based on predictions of expected effects and available resources (i.e., time and costs) [158]. Available measurement options and equipment need to be taken into consideration; and a mix of qualitative (collected through asking questions in the consultation) and quantitative data (collected from self-monitoring, computer programs/apps, telephone or electronic follow-up) is recommended [158]. Lastly, monitoring and evaluation outcomes should be fed back and shared with all HCPs treating the patient to raise the success rate in achieving the patient’s desired health outcomes and to justify the importance of adequate nutrition and the dietitian’s role in health care.

## 8. Hospital-to-Community Transition

Transitioning home from hospital is a critical step in the management of malnutrition, frailty and sarcopenia in community-dwelling adults. Rehospitalization rates for older adults are high and up to a third of readmissions are considered preventable [161]. Providing quality nutrition care upon and/or after discharge has been shown to reduce avoidable readmissions by 28% [161], due to the effect that nutrition intervention (predominately, prescription of ONS) has on oral intake and nutritional status [147,148,164,165,166]. Despite this, many older adults who are in need of quality nutrition care in this transition period do not receive it [167]. Communication problems between HCPs and across healthcare services, insufficient knowledge/attention to nutritional needs/problems by HCPs, and limited access to services have been described as barriers to delivering continuity of care [168,169,170]. Further complicating the problem is low compliance among those patients who do receive this care [153,164]. Qualitative and observational work has shown that gastrointestinal symptoms, lack of knowledge of ONS purpose, lack of ONS prescription and receiving nutrition care that lacked a person-centred approach are common reasons for low compliance among patients [153,171]. These findings demonstrate that the nutrition care of older adults during hospital to home transition periods needs improvement.

## 9. Conclusions 

While the different populations, definitions and diagnostic criteria used by individual studies may explain the large variance in prevalence rates reported for these conditions, it is clear that malnutrition, frailty and sarcopenia affect a large proportion (~25%) of community dwelling adults. Given our ageing population, it is important for health professionals to be proficient in recognising and treating malnutrition, frailty and sarcopenia in the community. The NCP should be used to guide HCPs in providing consistent and high-quality nutrition care for these conditions, using tools and approaches appropriate for each step: (i) screening and referral, (ii) assessment and diagnosis, (iii) intervention and (iv) monitoring and evaluation. Further, the transition of patients from hospital to home, or between care settings, should be considered in any care plan for community-dwelling adults. While this paper provides broad evidence-based recommendations (Table 6), next steps in this area should include: (i) exploration of the barriers limiting the delivery of high-quality care to older adults in the community who are malnourished, frail or sarcopenic (or are at risk of these conditions), from the perspectives of consumers and HCPs; and (ii) development of specific guidance for HCPs to manage and treat these conditions in the community, considering this is currently non-existent. 

## Figures and Tables

**Table 1:** Risk factors for malnutrition, frailty and sarcopenia in older community-dwelling adults.

Domain	Malnutrition	Frailty	Sarcopenia
Nutritional	Poor appetite [12,66,67] Poor dentition [67] Dysphagia [36,66] Low intake of milk/milk alternatives [32,33] Food avoidance [33] Eating alone [32] Finding meal preparation a chore [33]	Being malnourished [68] Low protein intake [68,69] Poor diet quality [68] Poor dietary antioxidant intake [40,68] Higher DII® scores [70] Low number of teeth [71] Poor masticatory function [71]	Low protein intake [72] Poor diet quality [73]
Physical function and form	Lower BMI/BW [36] Eating dependency [12,66] Poor physical function [12,34,66] Difficulty walking/climbing stairs [31] Unhealthy gait speed [36] Perceiving weight more than is [32,33]	Higher BMI [15,42,74]	Sedentary behaviour [75]
Psychosocial	Living alone, widowed, divorced, separated or single [31,34,76] Dementia / cognitive decline [66] Loss of interest in life [66] Depression [34]	Widowed, divorced or never married [16]	
Disease and care	Hospitalization [12,31,67] Parkinson disease [66] Constipation [66] Having no diabetes [67] Poor self-reported health [12,66,67] Polypharmacy [66]	Hospitalization [41] Multimorbidity [15,42,44] Polypharmacy [39,44,77] High mean DBI [39,77] More likely to have visited a HCP prior to a problem [41]	
Demographic	Increasing age [31,36,66] Low income level [76] Low educational level [34,76] Women [34]	Increasing age [15,16,42,44] Women [16,42,44] Low educational level [44] Men [15]	Increasing age [44,47]

ANZ: Australian and New Zealand; BMI: Body mass index; BW: body weight; DBI: Drug Burden Index; DII®: Dietary Inflammatory Index; HCP: health care professional. Only findings generated from original ANZ studies and reviews of international literature are summarised. Note: References [12,66,67,68,69,70,71,72,73] are reviews of international literature.

**Table 2:** Malnutrition, frailty and sarcopenia screening tools—validity in community settings.

Name		Description	Validity in Community Setting	Recommendation/Comment
Malnutrition screening tools				
Determine your Health Checklist (DETERMINE)		Self-completed, 10-question survey assessing dietary intake, nutrition impact symptoms, health conditions, medications, social/economic factors, weight changes and functional status [82].	Reported criterion validity show 75–91% sensitivity and 11–54% specificity; however few studies used appropriate reference standards [83].	Designed to assess nutritional status among community-dwelling older adults [82]; however predictive validity in community setting is poor (unable to predict mortality, hospitalisation, or weight loss of >5%).
Malnutrition Universal Screening Tool (MUST)		Considers BMI, weight loss and acute disease effect.	Two validation studies in community: 100% sensitivity, 98% specificity when validated against dietitian assessment; 58% sensitivity, 96% specificity when validated against unintentional weight loss or BMI [83].	Has been validated in hospital, residential aged care and community settings [84]; but more validation studies are needed in community.
Mini Nutritional Assessment-short form (MNA-SF)		Six items on dietary intake, weight loss, mobility, disease/stress, neurological problems and BMI [85].	Promising criterion validity in community setting, with high sensitivity (81–100%) and specificity (82–100%); however, studies used MNA-FF as reference standard so incorporation bias is present [83].	Recommended for use with older adults and validated in hospital, residential aged care and community settings.
Malnutrition Screening Tool (MST)		Two questions on appetite and unintentional weight loss [86].	Widely validated in hospital settings, with high sensitivity (90–98%) and specificity (85–89%); but not validated in community [83].	Community validation studies needed.
Seniors in the Community: Risk Evaluation for Eating and Nutrition Questionnaire (SCREEN-II; now called SCREEN-14)		Considers weight, appetite, dietary intake, nutrition impact symptoms, ONS intake, social factors [87].	Good validity among older community-dwelling Canadian and New Zealand adults, with reported sensitivity from 84–90% and specificity 62–86% when tested against clinical assessment by a trained dietitian. More validation studies needed in other settings [83].	Was developed to assess general nutrition status in community-dwelling older adults, but is also validated as a malnutrition screening tool [88].
**Frailty screening/assessment tools ^**				
The abbreviated Comprehensive Geriatric Assessment (aCGA)	15 questions on functional status, cognitive status and depression [89].		Good sensitivity (75–88%), moderate specificity (48–60%) for predicting functional decline/disability, mortality and institutionalisation in community-dwelling adults [90].	Acceptable performance for predicting disability only, and not related to mortality or institutionalization; therefore, not recommended as first choice for screening.
FRAIL scale	Self-administered survey on ambulation, fatigue, illness, resistance and weight.		Using Fried frailty phenotype as a reference standard, sensitivity was 87–96% and specificity 64–86%, with a FRAIL scale score of 2 being the optimal cut-off point, among community-dwelling Australians [91] and Chinese [92].	Good validity in community setting. A strength is that it does not require measurements nor administration by healthcare professionals [17,93].
Frailty Index	Underpinned by biological causative theory, evaluates health deficits (comorbidities, symptoms, disabilities, diseases).		Adequately predicts adverse health outcomes and correlates strongly with other frailty measures. Sensitivity is 46–61% and specificity 84–90% when compared with Fried’s Frailty Phenotype [94].	Considered gold standard for frailty screening due to high validity and ability to predict cause-specific mortality; however can be complex and time consuming to complete due to its mathematical nature, reducing its popularity clinically [17].
Fried’s Frailty Phenotype (also known as Fried Scale)	Underpinned by biological causative theory and considers weight loss, exhaustion, grip strength, gait speed and physical activity [13].		Can identify frailty and predict adverse clinical outcomes; hence is widely used in clinical and research settings. Low-moderate sensitivity (40–44%) and high specificity (85–94%) for predicting functional decline/ disability, mortality and institutionalisation in community-dwelling adults [90].	Requires measurement of handgrip strength and gait speed, which are not always practical/feasible in community settings.
Gérontopôle Frailty Screening Tool	Involves two steps: questionnaire evaluating weight, exhaustion, slowness, cognition, dependence; and clinician judgement of frailty.		Good sensitivity (88%) and specificity (84%) when assessed against Cardiovascular Health Study criteria definition of frailty as reference standard [95]. Considered one of the most appropriate frailty screening tools for use in community-dwelling adults.	Designed for early frailty recognition in community-dwelling older people [96]; however, its lack of specific guidance for clinicians to identify frailty is a limitation [97].
Groningen Frailty Indicator	15 items covering physical, cognitive, social and psychological domains [98].		Moderate sensitivity (52–63%) and specificity (69–77%) for predicting functional decline/disability, mortality and institutionalisation in community-dwelling adults [90].	Can determine the level of frailty.
Tilburg Frailty Indicator	Self-administered 15-item questionnaire considering health, weight, walking difficulty, balance, hearing, sight, grip strength, fatigue, memory, sensory, anxiety, coping capacity, solitude and social support [99,100].		Moderate to good predictive validity for disability (35–87% sensitivity; 61-89% specificity), needing residential care (81–86% sensitivity; 59–62% specificity) and hospitalisation (33–65% sensitivity; 60–86% specificity) in a range of community-dwelling, older adult populations [100,101,102,103,104].	Developed for identifying frail community-dwelling older people, and is validated in this setting with high diagnostic accuracy (95% sensitivity; 86% specificity) for frailty [105]. A limitation is that it takes 14 mins to complete.
Vulnerable Elders Survey	Contains 13 questions on age, self-rated health, physical fitness and independence [106].		Considered the most suitable tool to predict functional decline/disability, mortality and institutionalisation in community-dwelling adults, with high sensitivity (88–92%) but low-moderate specificity (47–59%) for predicting these outcomes [90].	Developed to identify community-dwelling vulnerable elderly at risk for functional decline. Relatively short and easy to complete.
Vulnerable Elders Survey	Contains 13 questions on age, self-rated health, physical fitness and independence [106].		Considered the most suitable tool to predict functional decline/disability, mortality and institutionalisation in community-dwelling adults, with high sensitivity (88–92%) but low-moderate specificity (47–59%) for predicting these outcomes [90].	Developed to identify community-dwelling vulnerable elderly at risk for functional decline. Relatively short and easy to complete.
**Sarcopenia screening tools**				
European Working Group on Sarcopenia in Older People (EWGSOP)	Two-step algorithm assessing gait speed and handgrip strength to identify people ‘at risk’ of sarcopenia who should then proceed to a full assessment [22].		Reported sensitivity is between 33–71% and specificity 89–91% when validated against multiple criteria for diagnosing sarcopenia [107]. *	Highly specific, relatively simple tool (however requires measurement of gait speed and handgrip strength). Cut-off thresholds for skeletal muscle mass indexes are 9.2 kg/m^2^ and 7.4 kg/m^2^ and for hand grip-strength are 32 kg and 22 kg, for males and females, respectively [108].
Goodman et al.’s screening grid	Assesses age and BMI to determine probability of low muscle mass [109].		Reported sensitivity is between 41–67% and specificity 86–89% when validated against multiple criteria for diagnosing sarcopenia [107]. *	Simple tool requiring only age and BMI. Individuals with a probability >70% in men and >80% in women considered at risk of sarcopenia [109]; however Locquet et al. found great variation in cut-offs across different definitions (17–73%) [107].
Ishii et al.’s score chart	Age, handgrip strength and calf circumference are used in a gender-adjusted equation to determine the probability of sarcopenia [110].		Reported sensitivity is between 84–100% and specificity 74–81% when validated against multiple criteria for diagnosing sarcopenia [107]. *	Excellent performance in identifying sarcopenia risk; however, involves complex and time-consuming calculations, limiting its practicability. Diagnostic cut-off scores (recommended by Locquet et al.) are 111.1 for men and 128.5 for women [107].
Mini Sarcopenia Risk Assessment (MSRA)	Considers age, hospitalisation in past year, activity level, number of meals/day, daily dairy intake, daily calorie intake, and weight loss in past year [111].		Reported sensitivity is between 78–90% and reported specificity is between 38–71%. MSRA-5 consistently has higher specificity than MSRA-7 [111,112].	Two versions; a five-item (MSRA-5) and a seven-item tool (MSRA-7). MSRA-5 is recommended due to easier completion and higher specificity than MSRA-7. Cut-offs to identify people with sarcopenia are 30 (MSRA-7) and 45 (MSRA-5).
The Strength, Assistance with walking, Rise from a chair, Climb stairs, and Falls (SARC-F)	Questionnaire on ability to carry a heavy load, walking, rising from a chair, climbing stairs, and frequency of falls [113].		Initially validated in community-dwelling Chinese adults with low sensitivity but good specificity [113]. Other studies report sensitivities between 36–56% and specificity between 85–87% when validated against multiple criteria for diagnosing sarcopenia [107]. *	Relatively simple to complete and score; but requires assessment on stairs and of lifting a 4.5 kg load. Two versions; SARC-F-5 and SARC-F-3, comprising five and three questions, respectively. SARC-F-5 is recommended due to better diagnostic performance. Score of ≥4 (out of 10) on SARC-F-5 indicates sarcopenia risk.
Yu et al.’s prediction equation.	Uses weight, BMI, age and sex to identify people ‘at risk’ of sarcopenia [114].		Reported sensitivity is between 16–83% and specificity 60–87% when validated against multiple criteria for diagnosing sarcopenia [107]. *	Recommended as a ‘rule-out’ screening test for sarcopenia (i.e., to reduce the number of costly DXA assessments undertaken). Cut-off values are dependent on the importance of DXA assessment cost vs. risk of missing sarcopenia cases [114].

DXA: Dual-energy X-ray absorptiometry ^ There is limited to no distinction between frailty screening and assessment tools; rather, tools are used interchangeably for both screening and assessment/diagnosis. * Sarcopenia screening tools were validated against multiple criteria for diagnosing sarcopenia: European Working Group on Sarcopenia in Older People (EWGSOP) [22], International Working Group on Sarcopenia (IWGS) [115], Asian Working Group for Sarcopenia (AWGS) [116], and Society for Sarcopenia, Cachexia and Wasting Disorders (SCWD) [117].

**Table 3:** Nutrition assessment domains and tools (partially adapted from NCP [81]).

Nutrition Assessment Domains	
Domain *	Description/Measures
Food/nutrition-related history	Adequacy of food/nutrition intake (via diet history), with consideration of medications, complementary/alternative supplements, nutritional supplements, nutrition knowledge/beliefs, food access/availability, physical activity. Low/reduced food intake may indicate malnutrition.
Anthropometric measurements	Height, weight, body mass index (BMI), weight history/weight change. Low BMI or unintentional weight loss may indicate malnutrition.
Biochemical data/test results	Blood laboratory results (e.g., electrolytes, iron), clinical tests (e.g., gastric emptying time, metabolic rate). Abnormal blood or clinical results may indicate malnutrition/risk; but should be considered alongside other domains.
Nutrition-focused physical findings	Physical appearance, appetite and other symptoms (swallow function, taste/smell changes, physical limitations). Thin appearance, muscle/fat wasting, reduced function, poor appetite and other nutrition impacting symptoms may indicate malnutrition.
**Frailty assessment domains**	
**Domain**	**Description/measures**
Health	Co-morbidities/illnesses, age, self-reported health status, recent hospitalisation, polypharmacy, symptoms.
Physical	Measures of weakness, exhaustion, decreased endurance/performance, slowness, balance, walking difficulty; weight loss, functional status, physical activity, dependence, disability (e.g., loss of hearing, sight).
Nutritional	Appetite, dietary intake, nutrition impacting symptoms.
Psychological	Cognition (memory, decision making), depression, anxiety.
Social	Coping capacity, solitude, social relations/support.
**Sarcopenia assessment domains**	
**Domain**	**Description/measures**
Health	Age, gender, recent hospitalisation
Physical	Physical activity, muscle quantity and function, strength, gait, falls
Nutritional	Weight, BMI, dietary intake, weight loss

* Other domains routinely assessed include: patient history (personal, medical, social history); nutrition assessment tools; aetiology category (nutrition diagnosis); and evaluation of progress towards nutrition-related goals/resolution of nutrition diagnosis(es).

**Table 4:** Summary of nutrition-related intervention studies targeting malnutrition, frailty or sarcopenia among community dwelling older ANZ adults *.

Study, Country	Study Design	Sample and Setting	Intervention	Assessment	Outcomes (Intervention vs. Control)
Malnutrition					
Leggo et al., 2008 [135]	Pre/post intervention	1145 adults (76.5 ± 9.2 years; 31% male) recruited from 16 Australian organisations caring for HACC clients in Australia	Adults identified as ‘at risk’ or ‘malnourished’ provided with at home, one-on-one individualized nutrition counselling from a dietitian for 6 months (median)	MST, PG-SGA	Of the 15% at risk/malnourished, 44% agreed to dietetic referral Nutrition status increased following intervention among the 34 patients followed up (82% had improved and 50% became well-nourished)
Hamirudin et al., 2016 [136]	Mixed-method pre/post intervention	143 adults (≥75 years; % male NS) recruited from 3 General Practices in NSW, Australia	Adults identified as ‘at risk’ or ‘malnourished’ provided with a resource kit ^a^ + other interventions (e.g., dietitian referral) by practice nurses for 6/12 months	MNA-SF, interviews	31% of adults at risk/malnourished at initial screen MNA-SF scores significantly improved from 9.9 ± 5.1 to 11.4 ± 2.1 in intervention group, while MNA-SF scores in the control group declined from 13.3 ± 0.9 to 12 ± 1.5
Hamirudin et al., 2017 [137]	Pre/post intervention	68 adults (85.5 ± 5.8 years; 47% male) recruited within 2 weeks post-discharge from hospitals in regional NSW, Australia	All adults provided with tailored individual dietary advice ^b^ at home by a dietitian for 3 months	MNA, body weight, BMI, diet history, food frequency checklist	Proportion of patients at risk/malnourished reduced from 61.8% at baseline to 23.5% at follow-up Mean body weight (67.1±13.5 kg to 68.0 ± 13.7 kg), MNA score (21.9 ± 3.5 vs. 25.2 ± 3.1) significantly improved pre/post Significant improvement in energy intake from ONS (+95.5 ± 388.2 kJ/day) and milk (+259.6 ± 659.8 kJ/day) 10.3% were receiving MOW at both time points
Charlton et al., 2013 [138]	Mixed-method pre/post pilot intervention	12 adults (81.3 ± 10.9 years; 58% male) recruited from two MOW services in NSW, Australia	Provision of high protein, high-energy snacks five times a week, in addition to their usual MOW order, for 1 month	MNA, body weight, BMI, 24h diet recall, food frequency checklist, interviews	Significant reduction in the proportion of adults at risk (17% to 8%) and malnourished (67% to 25%) Mean body weight and BMI increased by mean of 0.75 ± 0.80 kg and 0.78 ± 1.16 kg/m^2^, respectively Increased mean energy (+415 ± 1477 kJ /day) and protein (+7.2 ± 14.06 g/day) intakes
**Frailty**					
Cameron et al., 2013 [139]	RCT	216 adults meeting FFP criteria (83.3 ± 5.9 years; 32% male) recruited from 16 organisations caring for HACC clients in Australia	Provision of an individualised, multifactorial, interdisciplinary exercise and nutrition program ^d^ for 12 months	FFP, Short Physical Performance Battery	Frailty prevalence significantly reduced following intervention (absolute difference 14.7%) Physical status remained stable in intervention group and declined in control group
Milte et al., 2016 [140]	RCT	175 adults recovering from hip fracture (≥70 years; 23% male) recruited from 3 acute care and 1 rehabilitation setting in SA and NSW, Australia	Provision of an individualized exercise and nutrition program ^e^ and fortnightly dietitian visit to review dietary intake and modify strategies for 6 months	HRQoL, QALY, costs	Both groups saw a decrease in HRQoL score, but intervention group reported higher mean HRQoL Programme associated with a small additional cost and a gain in QALY relative to usual care with social visits

ANZ: Australian and New Zealand; BMI: Body mass index; FFP: Cardiovascular Health Study Frailty Phenotype; g: grams; HACC: Home and Community Care; HRQoL: Health-related quality of life; kJ: kilojoules; MNA (+/− SF): Mini Nutritional Assessment (+/− Short Form); MOW: Meals on Wheels; NSW: New South Wales; NS: not specified; PG-SGA: Patient-Generated Subjective Global Assessment; RCT: Randomised Control Trial; SA: South Australia; QALY: Quality adjusted life years. * Exercise is commonly used and is effectives in treating and managing frailty and/or sarcopenia in older adults; however, given the focus of this review, only interventions using nutrition-related strategies alone or in combination with exercise are included here. ^a^ Kit included: leaflet on high-energy/-protein foods, ‘Eating Well’ booklet, local council directory of nutrition/support services for older persons in their area. ^b^ Strategies included: personalised dietetic advice, prescription of ONS, referral to a MOW service and/or referral to various community services. ^c^ Intervention included: community dietitian and/or speech therapist consults, needs assessment and service coordination, day care and/or home delivered meals. ^d^ Nutrition program included: dietitian evaluation, home-delivered meals, ONS prescribed. ^e^ Nutriton program included: Dietary counselling focusing on timing, size, and frequency of meals, recommendations of nutrient-rich foods and recipes, referral to community meal programmes, and provision of commercial oral nutritional supplements or commercial protein powders as deemed appropriate.

**Table 5:** Outcome/indictor categories to assess nutrition intervention.

Category	Outcome/Indicator
1. Direct nutrition	Knowledge gained Behaviour change Food & nutrient intake Nutritional status
2. Clinical & health status	Biochemical data Weight/anthropometry Blood pressure Risk factor profile Disease signs and symptoms (e.g., muscle/fat wasting, appetite)
3. Patient value-based care	Quality of life Patient satisfaction Self-efficacy / self-management Functional ability
4. Healthcare utilisation/cost savings	Complications Medication changes Number of unplanned clinic visits Number of preventable hospital admissions Length of hospitalisation Nursing home admission

Adapted from Splett et al., 2001 [161] and Cant, 2008 [162].

**Table 6:** Recommendations for the nutritional management of malnutrition, frailty and sarcopenia.

Screening and Referral
Use a validated screening tool to identify patients at risk of malnutrition, frailty or sarcopenia. Tools that perform best in the community setting include: Malnutrition: SCREEN-14 (with MNA-SF and MUST also performing well) Frailty: Vulnerable Elders Survey (FRAIL scale, Fried’s Phenotype also perform well) Sarcopenia: MSRA-5 or SARC-F Tool selection should be based on performance in the intended setting (see Table 2) and with consideration of the time, resources and staffing/skill level required to complete.
Screening should be completed upon: Initial contact with a new patient Changes in a patient’s health status (e.g., new diagnosis or recent hospitalisation) Suspected malnutrition, frailty or sarcopenia (e.g., presence of risk factors)
If it is not possible to screen using a validated tool, HCPs should consider the patient’s risk factors (see Table 1) to determine if they are at risk / would benefit from a full assessment (e.g., by a dietitian or exercise physiologist).
Screening should be prioritised for patients who are older, have multiple comorbidities, have recently lost weight/are underweight, or who appear malnourished/frail/sarcopenic.
**Assessment and diagnosis**
Patients identified as ‘at-risk’ of malnutrition, frailty or sarcopenia should be referred to an appropriate health professional (e.g., dietitian, physiotherapist/exercise physiologist) for a full assessment and diagnosis, to inform nutrition and/or exercise interventions.
These health professionals should use appropriate assessment tools or criteria to diagnose malnutrition, frailty or sarcopenia; and document the level/stage (if applicable). For example *: Malnutrition: Mini Nutritional Assessment (MNA) Frailty: Fried Frailty Phenotype Sarcopenia: European Working Group on Sarcopenia in Older People (EWGSOP2)
If it is not possible to refer patients to appropriate allied health professionals, clinicians should use the domains in Table 3 to make an informal assessment, in order to guide care.
**Intervention**
A combination of nutrition- and exercise-based strategies should be adopted to manage malnutrition, frailty and sarcopenia. Common nutrition-based strategies used in combination include: Nutrition education and high energy, high protein / dietary fortification Nutrition education and oral nutrition supplements Nutrition education and home delivery meal services
Strategies should be tailored to the individual, factoring in their preferences, needs and context/geographical area.
Further work with consumers and HCPs should be undertaken to determine feasible and effective ways of improving nutrition among community-dwelling older adults in ANZ.
**Monitoring and evaluating**
HCPs should monitor/evaluate nutrition interventions using a combination of the indicators listed in Table 5, in conjunction with factoring in the indicators most important to patients to provide evidence-based, patient-centred care. This may include evaluating the following: Food & nutrient intake (direct nutrition), weight (clinical & health status) and patient satisfaction (patient value-based care); or Quality of life (patient value-based care), number of preventable hospital admissions (healthcare utilisation/cost savings), biochemical data (clinical & health status)
Qualitative and quantitative measures should be used to collect data, and, where available, validated tools.
Findings should be communicated to all HCPs caring for the individual, as well as to the patient themselves.

* Note: There is limited evidence for the diagnostic accuracy of malnutrition, frailty and sarcopenia assessment tools in the community setting. The authors have suggested these tools based on the evidence available at the time this review was undertaken; however more diagnostic accuracy studies are needed in the community setting for all three conditions and their respective assessment tools.

## Data Availability

N/A—this study did not report on any data (narrative review).

## Supplementary Materials

The following are available online at https://www.mdpi.com/article/10.3390/nu13072316/s1, Table S1: Malnutrition, frailty and sarcopenia prevalence and risk in community-living older adults^.
