# Supplementary material for: Identifying and Managing Malnutrition, Frailty and Sarcopenia in the Community: A Narrative Review
Source: Nutrients. 2021 Jul 5;13(7):2316. doi: 10.3390/nu13072316 (PMC8308465; doi:10.3390/nu13072316)
Supplement: Supplementary file 1 [file nutrients-13-02316-s001.zip › nutrients-1268111-supplementary.pdf]

Table S1. Malnutrition, frailty and sarcopenia prevalence and risk in community-living older adults^

Author, year	Setting and sample	Participants[a]	Screening tool(s)	Prevalence
**Malnutrition studies**				
Winter et al., 2013 [21]	Victoria, adults aged ≥ 75y	*N* = 225<br>48% men, 81.3 ± 4.3y	MNA®-SF	Malnourished: 1%<br>At risk: 16%
McElnay et al., 2012 [20]	Hawke's Bay, Māori and non-Māori adults aged ≥ 65y	*N* = 473<br>47% men, 74y	SCREEN© II	High risk: 32.8%<br>Moderate risk: 23.7%
Wham et al. (2011a) [22]	Auckland, adults aged 80–85y	*N*=51<br>29% men, 82.4 ± 1.7y	SCREEN© II	Mean score: 52.2 ± 6.7<br>High risk: 31%<br>Moderate/low risk: 69%
Wham et al. (2011b) [23]	Rotorua, Whakatane and Opotiki, Māori and non-Māori adults aged 75–85y	*N* = 108<br>44% men, 76.6 ± 1.8y (Maori); 85.2±0.6y (Non-Maori)	SCREEN© II	Score: 46.4 ± 5.8 (living alone)<br>Score: 50.3 ± 5.1 (living with others)<br>High risk: 52%<br>Moderate/low risk: 48%
Rist et al, 2012 [24]	Victoria, adults aged ≥ 65y	*N* = 235<br>47% men; 82 ± 7.0y	MNA®	At risk: 34.5%<br>Malnourished: 8.1%
Stricher et al, 2018 [25]	New Zealand*, Māori and non-Māori adults aged ≥ 65y	*N* = 209<br>48% men, 84.6 ± 0.5y	BMI < 20 kg/m^2 or ≥10% unintended weight loss	Malnourished: 17.2% (3y incidence)
Watson et al., 2010 [26]	Christchurch, adults aged ≥ 70y	*N* = 152<br>38% men, 79.5y	SCREEN©	At risk: 23%<br>High risk: 31%
Craven et al, 2018 [27]	Queensland, adults aged ≥ 65y	*N* = 77<br>60% men, 73.3 ± 5.1y	SCREEN© II, SF-12	High risk: 40%<br>Moderate risk: 26%
Wham, Maxted et al., 2015 [28]	The Northland and Bay of Plenty, adults aged 75–79y	*N* = 67<br>44% men, 77±1.5y	SCREEN© II	Score: 47.8 ± 5.3<br>High risk: 63%
North et al., 2018 [29]	Five North Island locations, Māori and non-Māori adults aged 80–90y	*N* = 653<br>63% men, 82(5)y[b] (Māori); 85(1)[b]y (Non-Māori)	SCREEN© II	At risk: 54%<br>High risk (Māori): 49%<br>High risk (non-Māori): 38%
Chatindiara et al., 2019 [30]	Auckland, Māori/Pacific and non-Māori adults aged ≥ 65y	*N* = 257<br>47% men, 79(7)y[b]	MNA®-SF	At risk: 11%<br>Malnourished: 1%
Isenring et al., 2013 [31]	Brisbane, adults aged ≥ 65y	*N* = 254<br>52% men, 74.3 ± 6.0y	MNA®-SF	At risk: 3.9% (1y incidence)
**Frailty studies**				
Gnjidic et al., 2012 [33]	Sydney, men aged ≥ 70y	N = 1662<br>100% men, 76.9 ± 5.4y	FFP	Frail: 9%<br>Pre-frail: 41%

Thompson et al., 2018 [11]	South Australia, adults aged ≥ 65y	*N* = 8804<br>14% men, 80 (79–82)y[b]	FFP	Frail: 21%; pre-frail: 48% (overall)<br>Frail: 6%; pre-frail: 41% (men)<br>Frail: 23%; pre-frail: 49% (women)
Das et al., 2019 [34]	Sydney, men aged ≥ 70y	*N* = 330<br>100% men, 81.4 ± 4.6y	FFP	Frail: 6.4% (3y incidence)<br>Pre-frail: 53% (3y incidence)
Dent et al., 2015 [35]	Rural South Australia, adults aged ≥65y	*N* = 1501<br>45% men, 75.9±7.9y	FI	Frail: 25% (overall)<br>Frail: 29% (women)<br>Frail: 21% (men)
Tembo et al., 2020 [36]	Gelong, adults aged ≥ 60y	*N* = 707<br>49% men, 71.0 (65.2–77.5)y[b]	FFP	Frail: 16% (overall)<br>Frail: 13%; pre-frail: 48% (men)<br>Frail: 18%; pre-frail: 54% (women)
Widagdo et al., 2015 [37]	South Australia, adults aged ≥ 70y	*N* = 2087<br>51% men, 78.2 ± 6.7y	FI, PFS, FFP, SFP	Frail: 35% (identified by 1 measure); 1% (by all measures).<br>Frail: 18% (FI); 49% (PFS); 2% (FFP); 9% (SFP)
**Frailty and sarcopenia studies**				
Thompson, Solomon et al., 2021 [38]	North-West Adelaide, adults aged ≥ 65y	*N* = 716<br>45% men, 74.1 ± 6.1y	FFP, EWGSOP1	Frail and sarcopenic: 3%<br>Frail: 16%<br>Sarcopenic: 4%
**Sarcopenia studies**				
Hirani et al., 2015 [39]	Sydney, men aged ≥ 70y	*N* = 1678<br>100% men, 77y	FNIH	Sarcopenia I: 14%<br>Sarcopenia II: 5%<br>Sarcopenia III: 4%
Sim et al., 2019 [40]	Perth, older women	*N* = 903<br>0% men, 79.9±2.6y	FNIH, FNIH2, EWGSOP1, EWGSOP2	Sarcopenia: 9% (FNIH); 12% (FNIH2); 24% (EWGSOP1); 11% (EWGSOP2)
Sui et al., 2021 [41, 42]	Geelong, adults aged ≥ 60y	*N* = 665<br>51% male, 70 (64–75)[b]y (women); 70 (66–78)[b]y (men)	EWGSOP1, EWGSOP2, FNIH	Sarcopenic men: 3% (EWGSOP), 1% (EWGSOP2), 1% (FNIH)<br>Sarcopenic women: 6 % (EWGSOP1), 2% (EWGSOP2), 4% FNIH
Villani et al., 2020 [43]	Queensland, adults aged ≥ 50y with T2DM	*N* = 87 (67% men)<br>71.2 ± 8.2y	EWGSOP1, EWGSOP2	Sarcopenia: 7% (EWGSOP1); 2% (EWGSOP2)

EWGSOP1: European Working Group on Sarcopenia in Older People; EWGSOP2: Adapted EWGSOP1; FFP: Modified Fried Frailty Phenotype; FI: Frailty Index; FNIH: United States Foundation for the National Institutes of Health; FNIH2: Adapted FNIH; SFP: Simplified Frailty Phenotype; MNA®-SF: Mini Nutritional Assessment Short Form; PFS: Prognostic Frailty Score; Sarcopenia I: low appendicular lean mass alone; Sarcopenia II: low ALM with weakness; Sarcopenia III: sarcopenia with weakness and poor gait speed. SCREEN©: Seniors in the Community Risk Evaluation for Eating and Nutrition questionnaire; SCREEN© II: Modified version of SCREEN©; y: years.

^ In Australia and New Zealand

* Data only extracted for New Zealand dataset

[a] Mean (+/- standard deviation) unless otherwise indicated: [b] Median (IQR).
